# Genome-wide QTL and eQTL analyses using Mendel

**DOI:** 10.1186/s12919-016-0037-6

**Published:** 2016-10-18

**Authors:** Hua Zhou, Jin Zhou, Tao Hu, Eric M. Sobel, Kenneth Lange

**Affiliations:** 1 Department of Biostatistics, University of California, Los Angeles, CA 90095 USA; 2 Bioinformatics Research Center, North Carolina State University, Raleigh, NC 27695 USA; 3 Division of Epidemiology and Biostatistics, Mel and Enid Zuckerman College of Public Health, Tucson, AZ 85721-0066 USA; 4 Department of Human Genetics, University of California, Los Angeles, CA 90095 USA; 5 Department of Biomathematics, University of California, Los Angeles, CA 90095 USA; 6 Department of Statistics, University of California, Los Angeles, CA 90095 USA

## Abstract

Pedigree genome-wide association studies (GWAS) (Option 29) in the current version of the Mendel software is an optimized subroutine for performing large-scale genome-wide quantitative trait locus (QTL) analysis. This analysis (a) works for random sample data, pedigree data, or a mix of both; (b) is highly efficient in both run time and memory requirement; (c) accommodates both univariate and multivariate traits; (d) works for autosomal and x-linked loci; (e) correctly deals with missing data in traits, covariates, and genotypes; (f) allows for covariate adjustment and constraints among parameters; (g) uses either theoretical or single nucleotide polymorphism (SNP)–based empirical kinship matrix for additive polygenic effects; (h) allows extra variance components such as dominant polygenic effects and household effects; (i) detects and reports outlier individuals and pedigrees; and (j) allows for robust estimation via the t-distribution. This paper assesses these capabilities on the genetics analysis workshop 19 (GAW19) sequencing data. We analyzed simulated and real phenotypes for both family and random sample data sets. For instance, when jointly testing the 8 longitudinally measured systolic blood pressure and diastolic blood pressure traits, it takes Mendel 78 min on a standard laptop computer to read, quality check, and analyze a data set with 849 individuals and 8.3 million SNPs. Genome-wide expression QTL analysis of 20,643 expression traits on 641 individuals with 8.3 million SNPs takes 30 h using 20 parallel runs on a cluster. Mendel is freely available at http://www.genetics.ucla.edu/software.

## Background

The classical variance component model has been a powerful tool for mapping quantitative trait loci (QTLs) in pedigrees. Polygenic effects are effectively modeled by introducing an additive genetic variance component operating on the kinship coefficient matrix. With unknown or dubious pedigree structure, global kinship coefficients can be accurately estimated from dense markers using either the genetic relationship matrix (GRM) or the method of moments. In GWAS (genome-wide association studies), the 2 alleles of a SNP (single nucleotide polymorphism) shift trait means and can be tested as a fixed effect. However, fitting a variance component model is computationally challenging, especially when it has to be done for a large number of markers. In the newly released version of the Mendel software [1], Option 29 implements an ultrafast score test for pedigree GWAS. Score tests require no additional iteration under the alternative model. Only SNPs with the most promising score-test *p* values are further subject to likelihood ratio testing, thus achieving a good compromise between speed and power for large-scale QTL analysis. In this paper, we demonstrate the capabilities of Mendel on the Genetic Analysis Workshop 19 (GAW19) sequencing data.

## Methods

QTL association mapping typically invokes the multivariate normal distribution to model the observed *T*-variate trait ***Y*** ∈ ℝ^*n* × *T*^ over a pedigree of $n$ individuals. The standard model [2] collects the means of the responses *vec*(***Y***) into a vector ***v*** and the corresponding covariances into a matrix **Σ** and represents the loglikelihood of a pedigree as

$$ L = -\frac{1}{2} \ln\ \det\ \boldsymbol{\Sigma} -\frac{1}{2}{\left[vec\left(\boldsymbol{Y}\right)-\boldsymbol{\upsilon} \right]}^t{\boldsymbol{\Sigma}}^{-1}\left[vec\left(\boldsymbol{Y}\right)-\boldsymbol{\upsilon} \right], $$

where the covariance matrix is typically parametrized as **Σ** = 2**Σ**_*a*_ ⊗ **Φ** + **Σ**_*d*_ ⊗ **Δ**_7_ + **Σ**_*h*_ ⊗ ***H*** + **Σ**_*e*_ ⊗ ***I***. Here **Φ** is the global kinship matrix capturing additive polygenic effects, and **Δ**_7_ is a condensed identity coefficient matrix capturing dominance genetic effects. For **Φ**, Mendel can use (a) the theoretical kinship matrix from provided pedigree structures; (b) SNP-based estimates for the kinship of pairs of people within each pedigree; or (c) SNP-based estimates for the entire global kinship matrix ignoring pedigree information. To estimate kinship coefficients from dense SNP data, Mendel employs either the GRM or the method of moments [3, 4]. The household effect matrix ***H*** has entries *h*_*ij*_ = 1 if individuals *i* and *j* are in the same household and 0 otherwise. Individual environmental contributions and trait measurement errors are incorporated via the identity matrix ***I***. QTL fixed effects are captured through the mean component ***υ*** = ***Aβ*** for some predictor matrix ***A*** and vector of regression coefficients ***β***. To test a SNP against a *T*-variate trait, ***A*** is augmented with *T* extra columns holding the allele counts at the SNP, and the corresponding regression coefficients are jointly tested for association [5]. For longitudinal measurements of covariates such as smoke, age, and blood pressure medication (BPmed), we may either assume time varying effect sizes or constrain their effect sizes at different time points to be the same. The latter tactic leads to a more parsimonious and interpretable model and can be easily enforced by setting appropriate parameter constraints in Mendel’s control file, which lists the user’s choice of model parameters. In Mendel, SNPs with the most impressive test score *p* values (top 10 by default) are further tested by the more accurate, but slower, likelihood ratio method, thus achieving a good compromise between speed and power for large-scale QTL analysis. We refer readers to our companion manuscript [6] for more model and implementation details.

## Results and discussion

### Family data

#### Size and power study using simulated traits (SIMPHEN.1–200)

The power to detect the 6 functional variants in the *MAP4* gene on chromosome 3 is evaluated from the 200 simulation replicates of the trivariate traits systolic blood pressure (SBP) and diastolic blood pressure (DBP). Type I errors are evaluated from the univariate Q1 trait, which does not involve a major gene. Our analysis includes covariates sex, age, BPmed, smoke, and their pairwise interactions, and uses the theoretical kinship matrix as the additive polygenic variance component. We constrain the covariate effects to be equal across 3 time points. Table 1 shows that the type I error is well controlled. Not surprisingly the power for detecting the 2 rare functional variants 3-47913455 and 3-47957741 is extremely low.

**Table 1:** Empirical power for testing trivariate DBP and SBP traits and empirical type I error for testing the univariate Q1, based on simulation data in files SIMPHEN.1–SIMPHEN.200

SNP	MAF	(DBP_1_, DBP_2_, DBP_3_)			(SBP_1_, SBP_2_, SBP_3_)			Q_1_
		*β*_DBP_	%Var	Power	*β*_SBP_	%Var	Power	Size
3-47913455	0.0049	−5.4633	0.0036	0.05 ± 0.02	−8.7001	0.0044	0.06 ± 0.02	0.06 ± 0.02
3-47956424	0.3777	−1.4951	0.0117	0.35 ± 0.03	−2.3810	0.0143	0.42 ± 0.03	0.03 ± 0.01
3-47957741	0.0016	−5.0841	0.0024	0.04 ± 0.01	−8.0964	0.0030	0.06 ± 0.02	0.06 ± 0.02
3-47957996	0.0301	−4.6435	0.0122	0.82 ± 0.03	−7.3946	0.0149	0.89 ± 0.02	0.05 ± 0.01
3-48040283	0.0318	−6.2235	0.0229	0.84 ± 0.03	−9.9107	0.0278	0.89 ± 0.02	0.05 ± 0.01
3-48040284	0.0131	−6.9531	0.0091	0.47 ± 0.04	−11.0726	0.0111	0.56 ± 0.06	0.04 ± 0.01

#### QTL analysis of the real, 8-variate phenotype (**DBP**_***i***_, **SBP**_***i***_, ***i*** = 1, 2, 3, 4)

Our analyses are based on the genotype calls for 959 individuals (464 directly sequenced and the rest imputed) provided in the chrNN-geno.csv.gz files. SBPs and DBPs measured at 4 time points are available for 1389 members from 20 extended families. The largest family contains 107 individuals; the smallest, 27. Genotypes at 8,348,674 SNPs were available on 959 of the individuals. We analyzed all SNPs and pedigrees together for the 8-variate trait (SBP_*i*_, DBP_*i*_, *i* = 1, 2, 3, 4). Our model includes covariates sex, age, BPmed, smoke, and their pairwise interactions, and we constrain the covariate effects to be equal across 4 time points. The log-likelihoods of the null model (no SNPs included) using the theoretical kinship, GRM within pedigrees, or GRM across all individuals are −11675.95, −11696.90, and −11698.71, respectively, indicating that the provided pedigree information captures additive genetic effects adequately. The results summarized below use the theoretical kinship matrix.

**Fig. 1:**
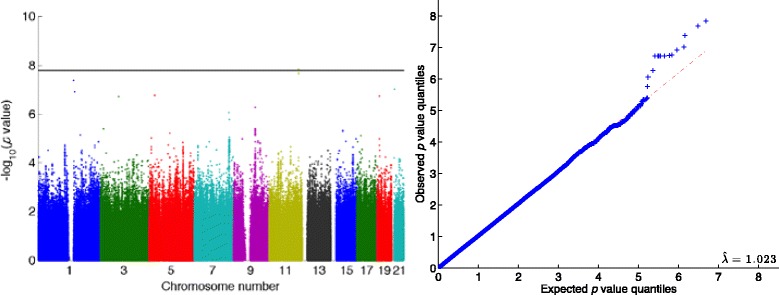
Multivariate QTL analysis of the real, 8-variate trait (SBP_*i*_, DBP_*i*_, *i* = 1, 2, 3, 4) from the family data with 849 individuals and 3.1 million SNPs (after filtering). Manhattan plot (*left*) and quantile–quantile (Q-Q) plot (*right*). The *horizontal line* represents the genome-wide significance level. The total run time on a laptop with an Intel Core i7 2.6 GHz CPU and 16 GB RAM was 78 min

To read in all the data and run standard quality control (QC) procedures took just under 5 min. QC excluded 10,603 SNPs and 110 individuals based on genotyping success rates below 98 %. The remaining 8,338,071 SNPs and 849 individuals were analyzed. The subsequent ped-GWAS analysis ran in 73 min for all results reported in Fig. 1 and Tables 2 and 3. Because we excluded rare SNPs with low minor allele frequencies <0.03 across 849 individuals, *p* values were calculated for only 3,084,046 SNPs. Accordingly the genome-wide significance threshold is 1.62 × 10^−8^ or 7.79 on the log_10_ scale; the threshold for a false discovery rate (FDR) of 0.05 is 4.19 × 10^−8^ or 7.38 on the log_10_ scale.

**Table 2:** Multivariate QTL analysis of the real, 8-variate trait (SBP_*i*_, DBP_*i*_, *i* = 1, 2, 3, 4) from the family data with 849 individuals and 3.1 million SNPs (after filtering). Estimated mean effects under the null model (no SNPs included) using the theoretical kinship matrix for the additive polygenic variance component

Mean effects	SBP_*si*_	DBPs_*i*_
*β*_Sex_	10.21	4.24
${\beta}_{{\mathrm{Age}}_i}$	0.32	0.02
${\beta}_{{\mathrm{BPmed}}_i}$	3.11	10.07
${\beta}_{{\mathrm{Smoke}}_i}$	1.53	1.84
${\beta}_{{\mathrm{Sex}}_i\times {\mathrm{BPmed}}_i}$	−3.20	−1.84
${\beta}_{{\mathrm{Sex}}_i\times {\mathrm{Smoke}}_i}$	0.30	−0.73
${\beta}_{{\mathrm{Sex}}_i\times {\mathrm{Age}}_i}$	0.41	0.14
${\beta}_{{\mathrm{BPmed}}_i\times {\mathrm{Smoke}}_i}$	3.83	2.43
${\beta}_{{\mathrm{BPmed}}_i\times {\mathrm{Age}}_i}$	0.01	−0.35
${\beta}_{{\mathrm{Smoke}}_i\times {\mathrm{Age}}_i}$	−0.06	−0.06

**Table 3:** Multivariate QTL analysis of the real, 8-variate trait (SBP_*i*_, DBP_*i*_, *i* = 1, 2, 3, 4) from the family data with 849 individuals and 3.1 million SNPs (after filtering). Three SNPs pass the FDR 0.05 threshold. The top SNP, 11-118783424, also passes the genome-wide significance level

SNP	Base pair	MAF	− log_10_(p)	HW *p* value
11-118783424	118,783,424	0.02778	7.84	0.7665
11-118767564	118,767,564	0.02778	7.68	0.7665
1-142617328	142,617,328	0.49074	7.38	0.0000

*HW* Hardy-Weinberg, *MAF* minor allele frequency

Table 2 lists the estimates for environmental effects and their interactions under the null model (no SNPs included). Figure 1 displays the Manhattan and quantile–quantile (Q-Q) plots. The genomic inflation factor of 1.023 indicates no systematic bias. One SNP passes the Bonferroni-corrected genome-wide significance level, and 3 SNPs pass the FDR 0.05 threshold. They are listed in Table 3. SNP 1-142617328 has a Hardy-Weinberg equilibrium (in founders) *p* <10^−22^, indicating possible genotyping error. The remaining 2 significant SNPs occur at 118,783,424 and 118,767,564 base pairs, respectively, on chromosome 11. Both show a minor allele frequency (MAF) of 0.02778 in 413 founders. Because the MAFs in all 849 individuals are higher than 0.03, they were not removed in the filtering stage.

#### Genome-wide expression QTL analysis of 20,634 expression traits

Genome-wide measures of 20,634 gene expression levels in peripheral blood mononuclear cells from the first study examination are provided for 643 individuals in the family data. The formidable task of exhaustive expression quantitative trait locus (eQTL) analysis (20,634 expressions vs. 8,338,071 SNPs) can be easily managed using Mendel. We submitted 20 parallel jobs to a cluster and finished the complete analysis in approximately 30 h.

**Fig. 2:**
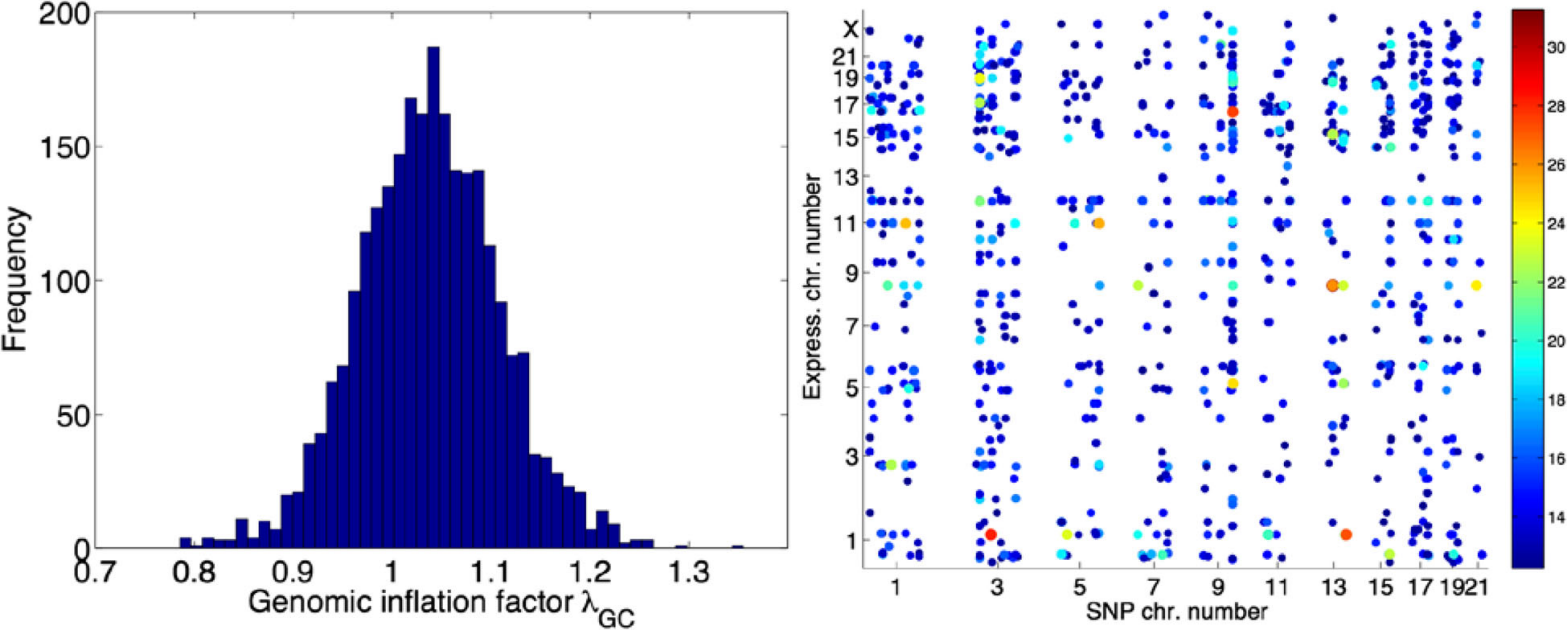
Summary of the eQTL analysis. *Left*: Histogram of the genomic inflation factors *λ*_*GC*_. *Right*: Top expression-SNP hits from the eQTL analysis. Each *dot* represents an expression-SNP association that satisfies: genomic inflation factor *λ*_*GC*_ < 1.1, *p* <5.77 × 10^−13^, SNP Hardy-Weinberg test (in founders) *p* >10^−8^, SNP MAF in 641 individuals >0.01, and the expression probe is annotated in the EXPR_MAP.csv file. Dot size and color vary according to their *p* values on the log_10_ scale. Total run time (20,634 expressions vs. 8,338,071 SNPs) on a cluster with 20 parallel jobs was approximately 30 h

In all eQTL runs, SNPs and individuals with genotyping success rate equal to or less than 0.98 are excluded from analysis. Rare variants with MAF $\mathrm{equal}\;\mathrm{t}\mathrm{o}\;\mathrm{o}\mathrm{r}\;\mathrm{less}\;\mathrm{t}\mathrm{han}\;0.01$ in all individuals are also excluded. This leaves 641 individuals and 4,199,714 SNPs. The theoretical kinship matrix is used for the additive polygenic variance component. Our analysis includes covariates sex, age, BPmed, smoke, and their pairwise interactions. Initialization takes approximately 5 min; the subsequent genome-wide QTL mapping of each expression trait takes approximately 1 to 2 min. The left panel of Fig. 2 displays a histogram of genomic inflation factors from 20,634 genome-wide QTL analyses. They are well-concentrated around 1 and indicate no or little systematic bias. The right panel shows the top hits that satisfy a set of stringent criteria listed in the figure caption. Note that the whole eQTL significance level is set at 0.05/20634/4199714 = 5.77 × 10^− 13^.

### Unrelated data

A second data set consists of exome sequence calls, blood pressure phenotypes at a single time point, and simulated phenotypes on a large set of unrelated individuals. Like the family data set, these individuals are Mexican Americans; however, they were independently ascertained and do not overlap with the family data set.

#### Size and power study using simulated traits (SIMPHEN.1–200)

The data set provides 200 simulation replicates of the trait SBPs and DBP. However, GAW19 organizers did not distribute the exact simulation details, except to state that “The set of causal variants is somewhat different since this is exome data rather than the full sequence data that was provided last time, and so not all of the GAW18 variants, regulatory ones in particular, are present in the new data set.” This precludes a precise size and power study.

For ease of comparison, we tested 5 of the 6 variants displayed in Table 1 (for family data) against the bivariate trait (SBP, DBP) for all 200 simulation replicates and report the rejection rates in Table 4. In the model, we include covariates sex, age, BPmed, smoke, and their pairwise interactions, and use the SNP-based genetic relation matrix for modeling additive polygenic inheritance.

**Table 4:** Empirical rejection rates (standard errors in parenthesis) for testing five variants in the *MAP4* gene against the bivariate (SBP, DBP) trait, based on simulation data in files SIMPHEN.1-SIMPHEN.200 for 1943 unrelated individuals

SNP	MAF	Rejection rate
3-47956424	0.3435	1.00 (0.00)
3-47957741	0.0005	0.09 (0.02)
3-47957996	0.0229	1.00 (0.00)
3-48040283	0.0281	1.00 (0.00)
3-48040284	0.0070	0.12 (0.02)

#### QTL analysis of the real, bivariate phenotypes (DBP and SBP)

**Fig. 3:**
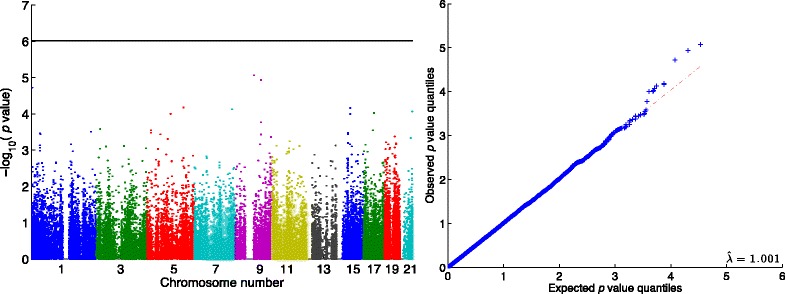
QTL analysis of the real, bivariate (SBP, DBP) trait for 1850 unrelated individuals and 52,314 SNPs with a MAF >0.01. Manhattan plot (*left*) and Q-Q plot (*right*). The *horizontal line* represents the genome-wide significance level; no SNPs pass this level. Total run time on a laptop with Intel Core i7 2.6 GHz CPU and 16 GB RAM was 39 min

The phenotypes SBP and DBP measured at the first examination are available for 1943 unrelated American Mexicans. We analyzed all SNPs and bivariate traits (SBP, DBP). To read in all the data and run standard QC procedures took 1 min and 16 s. QC excluded 10,191 SNPs and 93 individuals based on genotyping success rates below 98 %. The remaining 1,701,575 SNPs and 1850 individuals were analyzed. GRM calculated from whole genome SNPs was used to adjust for polygenic effects. The subsequent ped-GWAS analysis ran in 37 min and 5 s and included all of the results plotted in Fig. 3 and Table 5. Because we exclude rare variants with a MAF equal to or less than 0.01 in all individuals, *p* values were calculated for 52,314 SNPs. Accordingly, the genome-wide significance threshold is 9.56 × 10^−7^ or 6.02 on the log_10_ scale.

**Table 5:** QTL analysis of the real, bivariate (SBP, DBP) trait for 1850 unrelated individuals and 52,314 SNPs with MAF >0.01. Mean effects (standard errors in parenthesis) and variance components under the null model using GRM with all individuals

Mean effects	SBP	DBP
*μ*	94.87 (1.62)	78.46 (0.95)
*β*_Sex_	10.90 (1.63)	4.62 (0.95)
*β*_Age_	0.43 (0.05)	−0.13 (0.03)
*β*_Sex × age_	0.38 (0.06)	0.08 (0.04)
Var. comp.	$$ {\Sigma}_a=\left(\begin{array}{cc}\hfill 43.15\hfill & \hfill 17.03\hfill \\ {}\hfill 17.03\hfill & \hfill 12.07\hfill \end{array}\right) $$	$$ {\Sigma}_e=\left(\begin{array}{cc}\hfill 294.88\hfill & \hfill 113.90\hfill \\ {}\hfill 113.90\hfill & \hfill 102.61\hfill \end{array}\right) $$

Estimated environmental effects and their interactions and variance components under the null model (no SNPs included) are listed in Table 5. Figure 3 displays the Manhattan and Q-Q plots. The genomic inflation factor of 1.001 indicates no systematic bias. No SNPs pass the genome-wide significance level or FDR 0.05 threshold.

## Conclusions

All analyses in this article use Mendel v14.3, which is freely available at http://www.genetics.ucla.edu/software. Pedigree GWAS (Option 29) in Mendel proves to be an extremely efficient and versatile implementation for large-scale QTL analysis. Most competing programs ignore multivariate traits and outliers altogether. See Zhou et al [6] for a side-by-side comparison with the Factored Spectrally Transformed Linear Mixed Model (FaST-LMM) [7] and GEMMA (Genome-wide Efficient Mixed-Model Analysis) [8] programs. Here we have emphasized Mendel’s flexibility in specifying the global kinship matrix, adjusting for confounding, and capturing interactions. These assets, plus its raw speed, make it an ideal environment for QTL mapping. Mendel continues to mature, and geneticists are advised to give it a second look for genetic analysis [1]. In rare variant mapping, each variant may be too rare to achieve significance in hypothesis testing. Grouping related SNPs in a variance component may be more powerful than the mean component models used here. Extending Mendel to test variance component is among the focuses of our current work.
